# Supplementary material for: Generation of the transgene-free canker-resistant Citrus sinensis using Cas12a/crRNA ribonucleoprotein in the T0 generation
Source: Nat Commun. 2023 Jul 5;14:3957. doi: 10.1038/s41467-023-39714-9 (PMC10319737; doi:10.1038/s41467-023-39714-9)
Supplement: Supplementary file 1 — Supplementary Information [file 41467_2023_39714_MOESM1_ESM.pdf]

Generation of the transgene-free canker-resistant *Citrus sinensis* using Cas12a/crRNA ribonucleoprotein in the T0 generation

Su *et al.*

Supplementary Table 1. Summary of genomic sequencing of *CsLOB1*-edited *C. sinensis* cv. Hamlin lines generated by LbCas12aU RNP transformation of embryogenic citrus protoplasts.

**Types of regenerated lines**	**Raw data (Gb)**	**High quality data (Gb)**
L1	23.29	22.79
L2	22.56	22.35
L3	21.74	21.22
L4	17.23	16.91
L5	20.53	20.15
L6	23.50	23.07
L7	23.61	23.12
L8	27.81	27.14
L9	19.15	18.76
L10	18.53	18.24
L11	21.79	21.34
L12	21.51	21.09
Wild type	23.07	22.61

Supplementary Table 2. Primers, and crRNAs sequences.

Name	Sequences (5'-3')
F-LOB1-offtarget	TCAGCATGCGTTTTTGCTGT
R-LOB1-offtarget	CTTGATCTCCTCTACTTTGGGG
F-PDS-offtarget	GCATATAGCTCGCTCGGCAA
R-PDS-offtarget	CGTTGTCGTCAATGATTAACTCG
Primers for qRT-PCR	
F-Cs7g32410	GCCTCAGGAACAATGGGAGG
R-Cs7g32410	CCGTGTTAACGCCGTATCCT
F-Cs6g17190	CTCTCGCAGCTCCATTCTGT
R-Cs6g17190	GTCGCCGAACACCGATAAGA
F-1t00600	CTGGCGCTTCAACGATATGC
R-1t00600	GTGAGAGGTAGACGGCGAAG
F-Cs9g17380	CTTTGCAGTGGTGGCTCTTG
R-Cs9g17380	TTTGGTCAAGGCTCTCGCAT
F1-RT-LOB1	CCACCAACCGAACCATACAA
R1-RT-LOB1	CCATGCTGCTCACTGCATCT
F2-RT-LOB1	AAGGCACAGGCTGAGCTTGT
R2-RT-LOB1	AAGACTTGTTCTTGAGATTGTGCCA
F3-RT-LOB1	GGCACAATCTCAAGAACAAGTCT
R3-RT-LOB1	GGCTCCCAAGCTGATCCAAT
F-CsGAPDH	GGAAGGTCAAGATCGGAATCAA
R-CsGAPDH	CGTCCCTCTGCAAGATGACTCT
F-PR1b	GATGGGAAGCCATTATACGACTAC
R-PR1b	ACAAAGTTGAGAGTGCCATTGTTA
F-PR2	CCTTGTTCCCGCCATGAG
R-PR2	GCCAAGAGCTCCAGTTTCGA
F-PR5	ATTGCCAATAACCCTAATGAAAAA
R-PR5	GACAGTTACCGTTAAGATCAGCAA
crRNA sequences	
PDS (BccI)	CGAGATAGTGAACCGATGGGTCA
LOB1-3(BlpI)	CATGGTGACAAGCTCAGCCTGTG

Note: The restriction enzyme digestion sites were included in the parenthesis

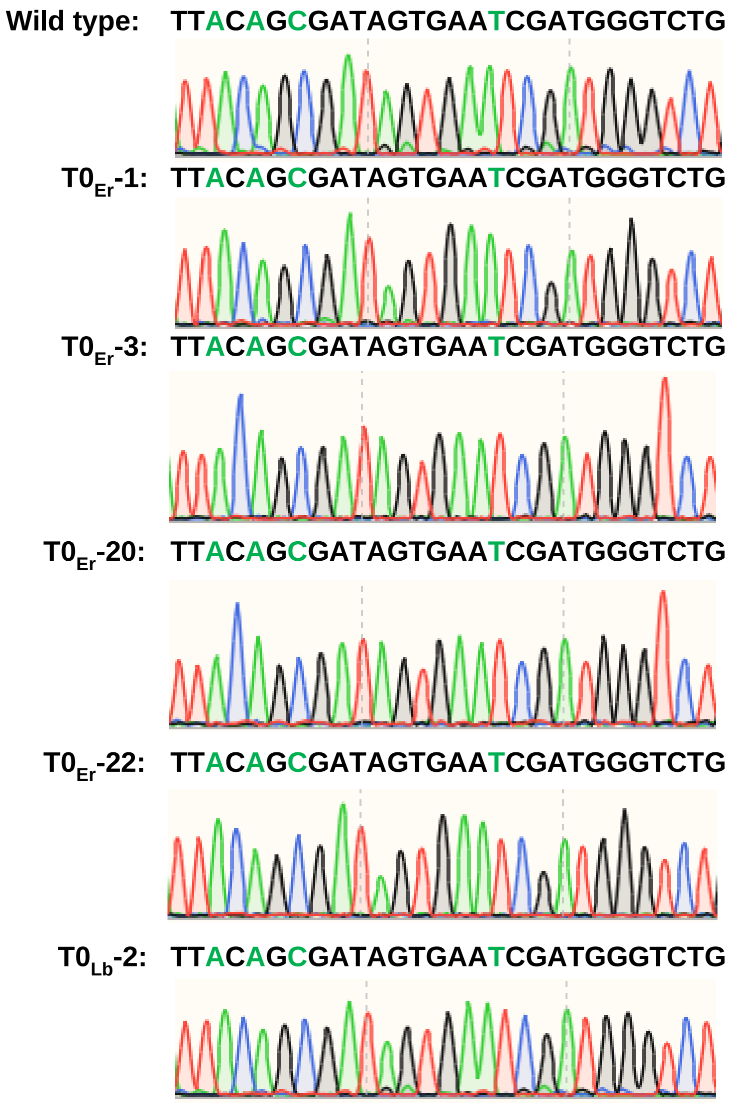

Supplementary Figure 1. Off-target analysis by PCR amplification, cloning and sequencing of embryos generated after ErCas12a and LbCas12aU RNP transformation. 58 embryos generated after ErCas12a RNP transformation of embryogenic protoplasts and 15 embryos generated after LbCas12aU RNA transformation of embryogenic protoplasts were tested. The sequencing result of representative embryos from each genotype were shown. No off-target activity was detected in these embryos.

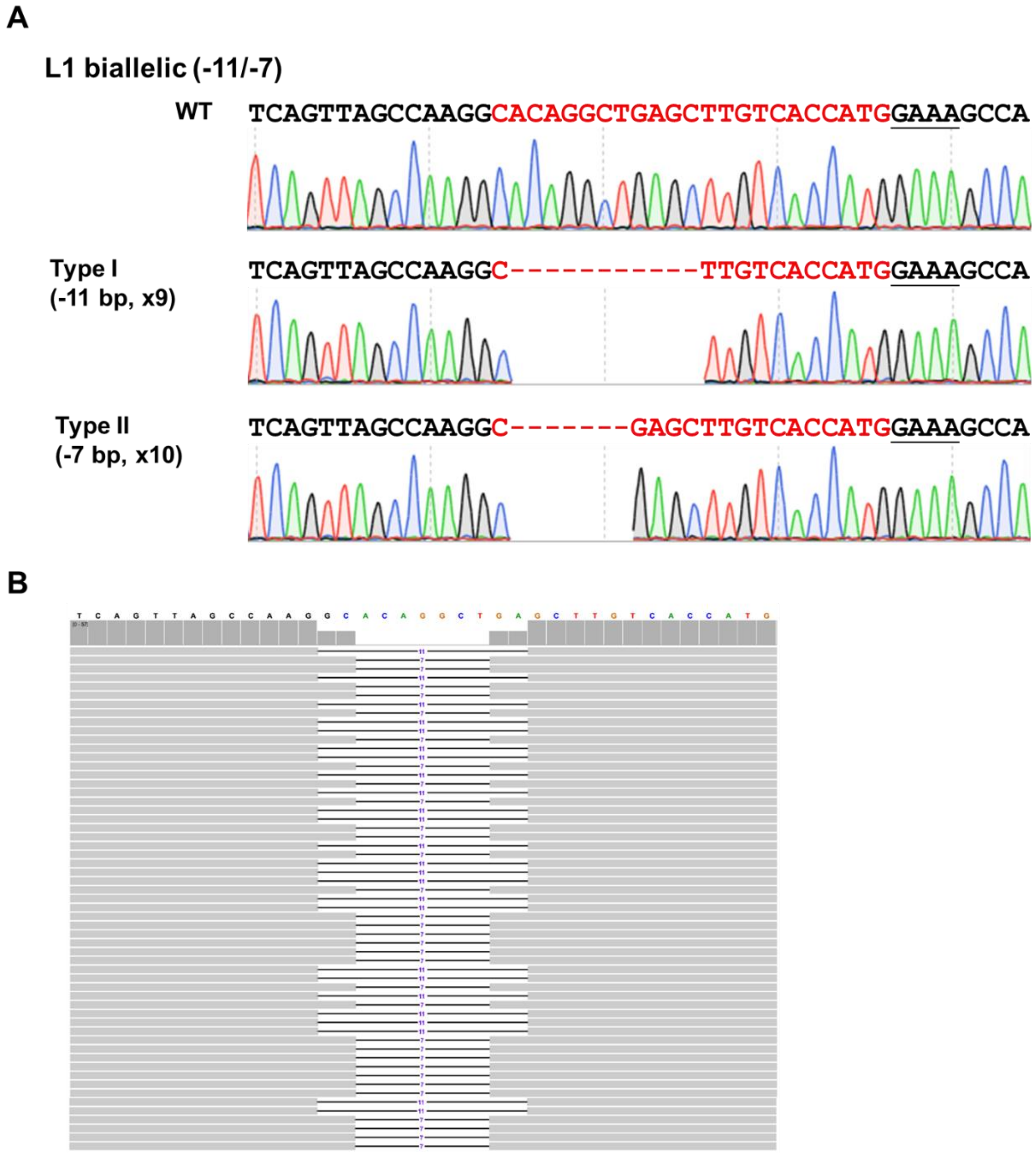

Supplementary Figure 2. Sequencing confirmation of the transgene-free *CsLOB1*-edited *C. sinensis* cv. Hamlin line L1 generated by LbCas12aU/crRNA RNP transformation of embryogenic citrus protoplasts. A. Sequencing confirmation of *CsLOB1* based on PCR amplification and cloning. The representative chromatograms of *CsLOB1* edited *C. sinensis* cv. Hamlin lines. The mutations of both alleles of *CsLOB1* were shown for each line. x indicates number of colonies sequenced. Nucleotide in red indicates crRNA. The underlined GAAA indicates protospacer-adjacent motif (PAM). -: deletion. +: insertion. B. Whole genome sequencing of the edited lines using next generation sequencing. The bases of target site were highlighted by colors other than black. There were two types of deletions of target site, including type I (-11 bp deletion) and Type II (-7 bp deletion), which were shown by horizontal bar chart. The vertical bar chart showed the sequence depth for each nucleotide.

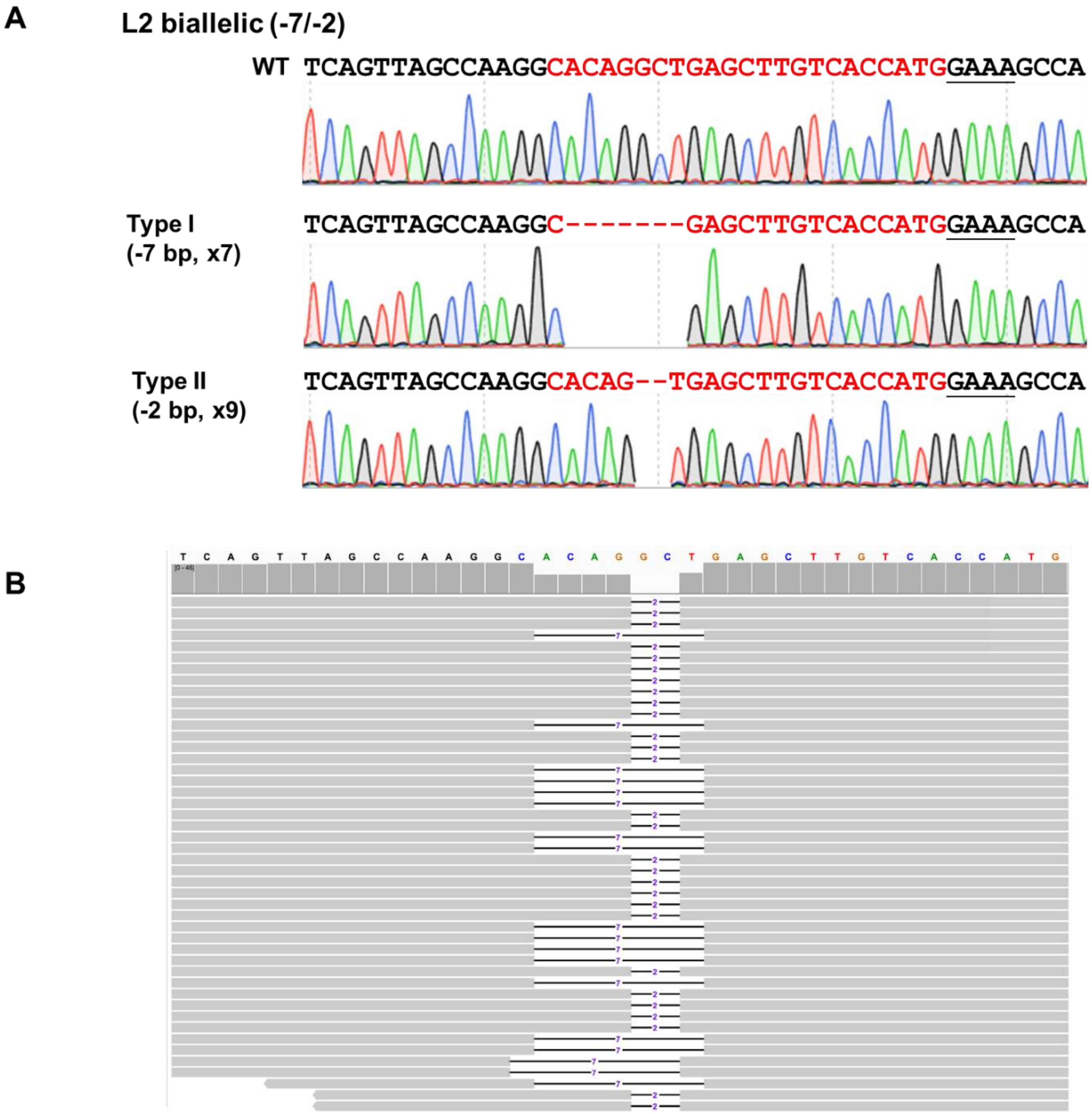

Supplementary Figure 3. Sequencing confirmation of the transgene-free *CsLOB1*-edited *C. sinensis* cv. Hamlin line L2 generated by LbCas12aU/crRNA RNP transformation of embryogenic citrus protoplasts. A. Sequencing confirmation of *CsLOB1* based on PCR amplification and cloning. The representative chromatograms of *CsLOB1* edited *C. sinensis* cv. Hamlin lines. The mutations of both alleles of *CsLOB1* were shown for each line. x indicates number of colonies sequenced. Nucleotide in red indicates crRNA. The underlined GAAA indicates protospacer-adjacent motif (PAM). -: deletion. +: insertion. B. Whole genome sequencing of the edited lines using next generation sequencing. The bases of target site were highlighted by colors other than black. There were two types of deletions of target site, including type I (-7 bp deletion) and Type II (-2 bp deletion), which were shown by horizontal bar chart. The vertical bar chart showed the sequence depth for each nucleotide.

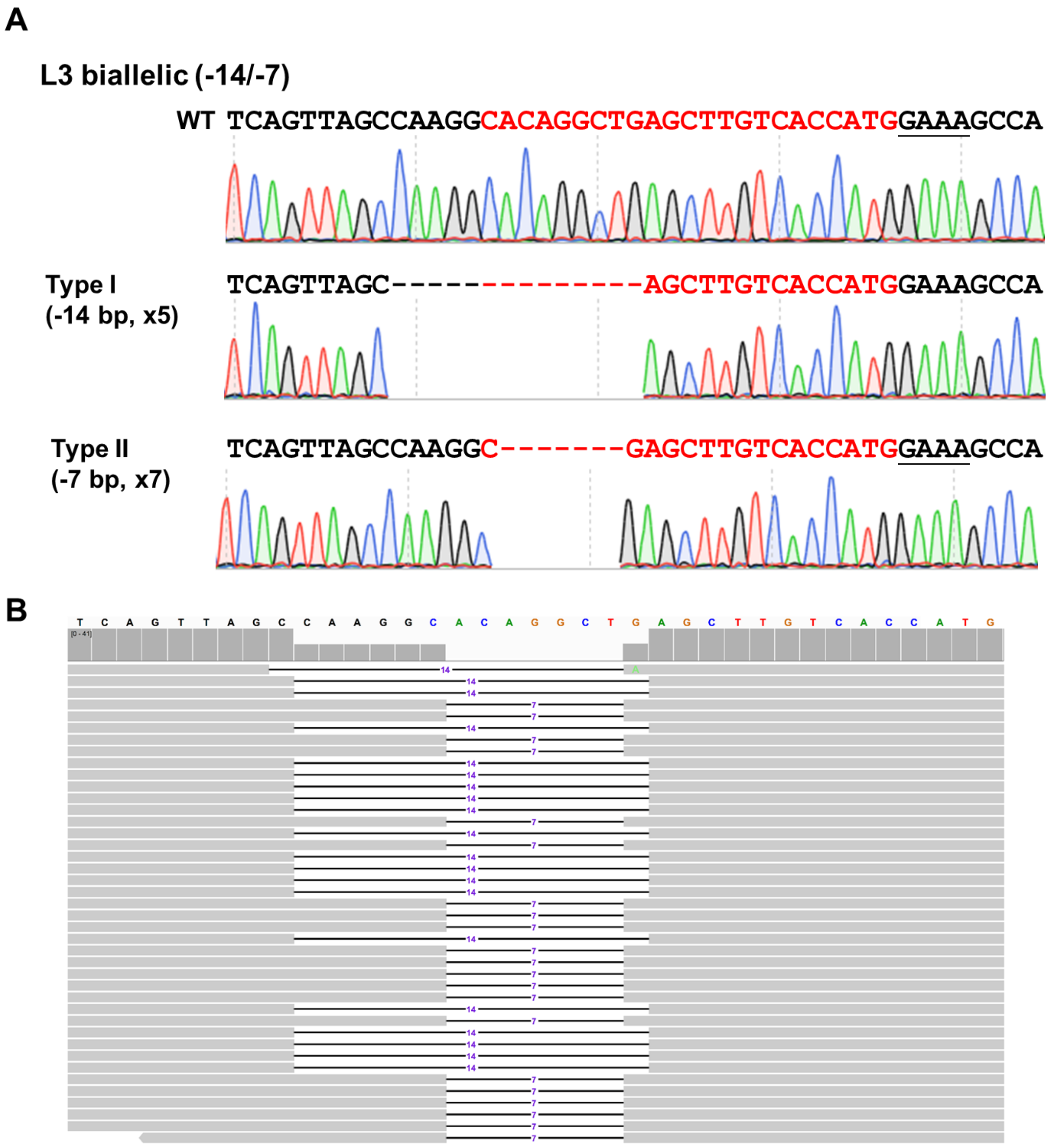

Supplementary Figure 4. Sequencing confirmation of the transgene-free *CsLOB1*-edited *C. sinensis* cv. Hamlin line L3 generated by LbCas12aU/crRNA RNP transformation of embryogenic citrus protoplasts. A. Sequencing confirmation of *CsLOB1* based on PCR amplification and cloning. The representative chromatograms of *CsLOB1* edited *C. sinensis* cv. Hamlin lines. The mutations of both alleles of *CsLOB1* were shown for each line. x indicates number of colonies sequenced. Nucleotide in red indicates crRNA. The underlined GAAA indicates protospacer-adjacent motif (PAM). -: deletion. +: insertion. B. Whole genome sequencing of the edited lines using next generation sequencing. The bases of target site were highlighted by colors other than black. There were two types of deletions of target site, including type I (-14 bp deletion) and Type II (-7 bp deletion), which were shown by horizontal bar chart. The vertical bar chart showed the sequence depth for each nucleotide.

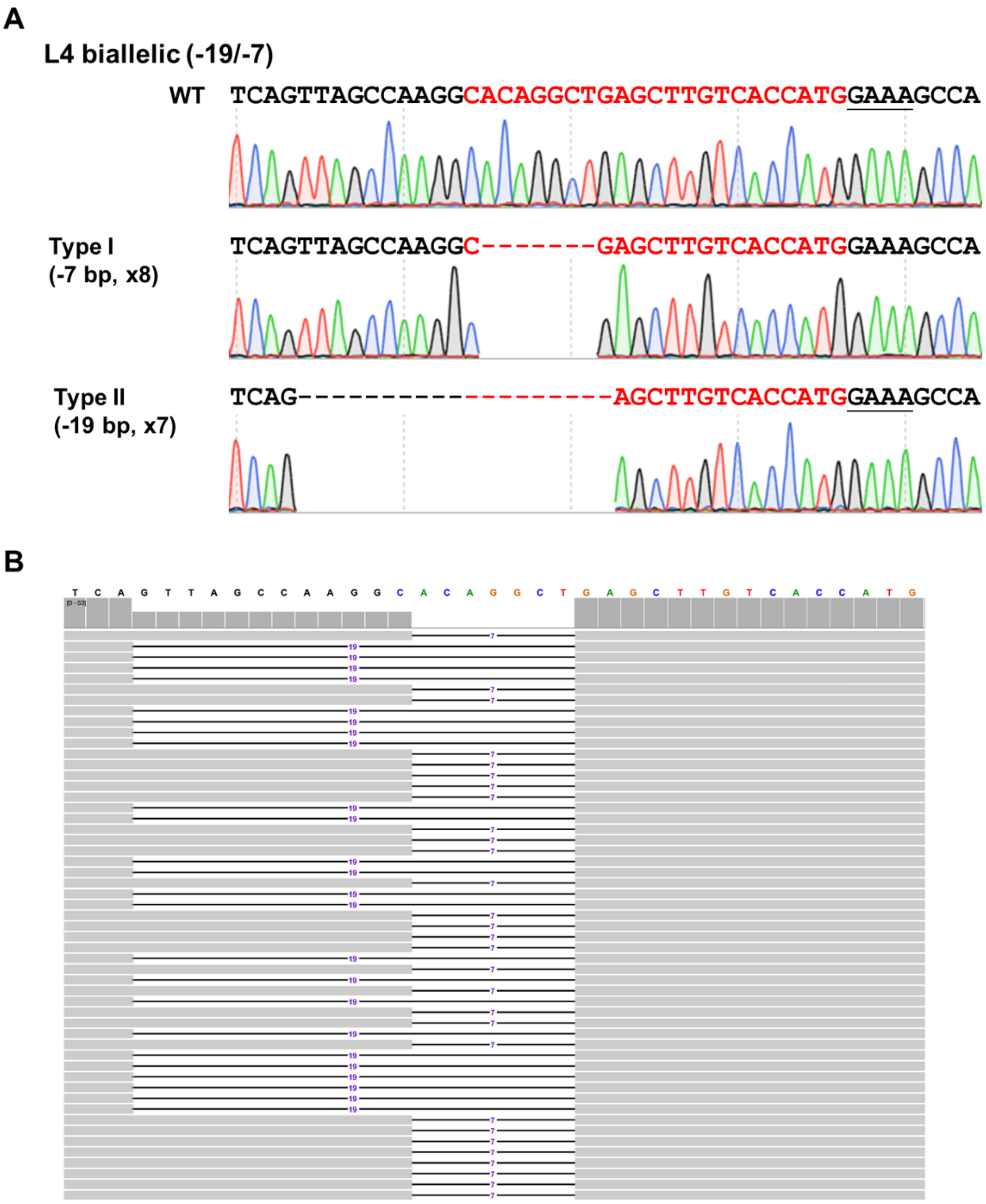

Supplementary Figure 5. Sequencing confirmation of the transgene-free *CsLOB1*-edited *C. sinensis* cv. Hamlin line L4 generated by LbCas12aU/crRNA RNP transformation of embryogenic citrus protoplasts. A. Sequencing confirmation of *CsLOB1* based on PCR amplification and cloning. The representative chromatograms of *CsLOB1* edited *C. sinensis* cv. Hamlin lines. The mutations of both alleles of *CsLOB1* were shown for each line. x indicates number of colonies sequenced. Nucleotide in red indicates crRNA. The underlined GAAA indicates protospacer-adjacent motif (PAM). -: deletion. +: insertion. B. Whole genome sequencing of the edited lines using next generation sequencing. The bases of target site were highlighted by colors other than black. There were two types of deletions of target site, including type I (-7 bp deletion) and Type II (-19 bp deletion), which were shown by horizontal bar chart. The vertical bar chart showed the sequence depth for each nucleotide.

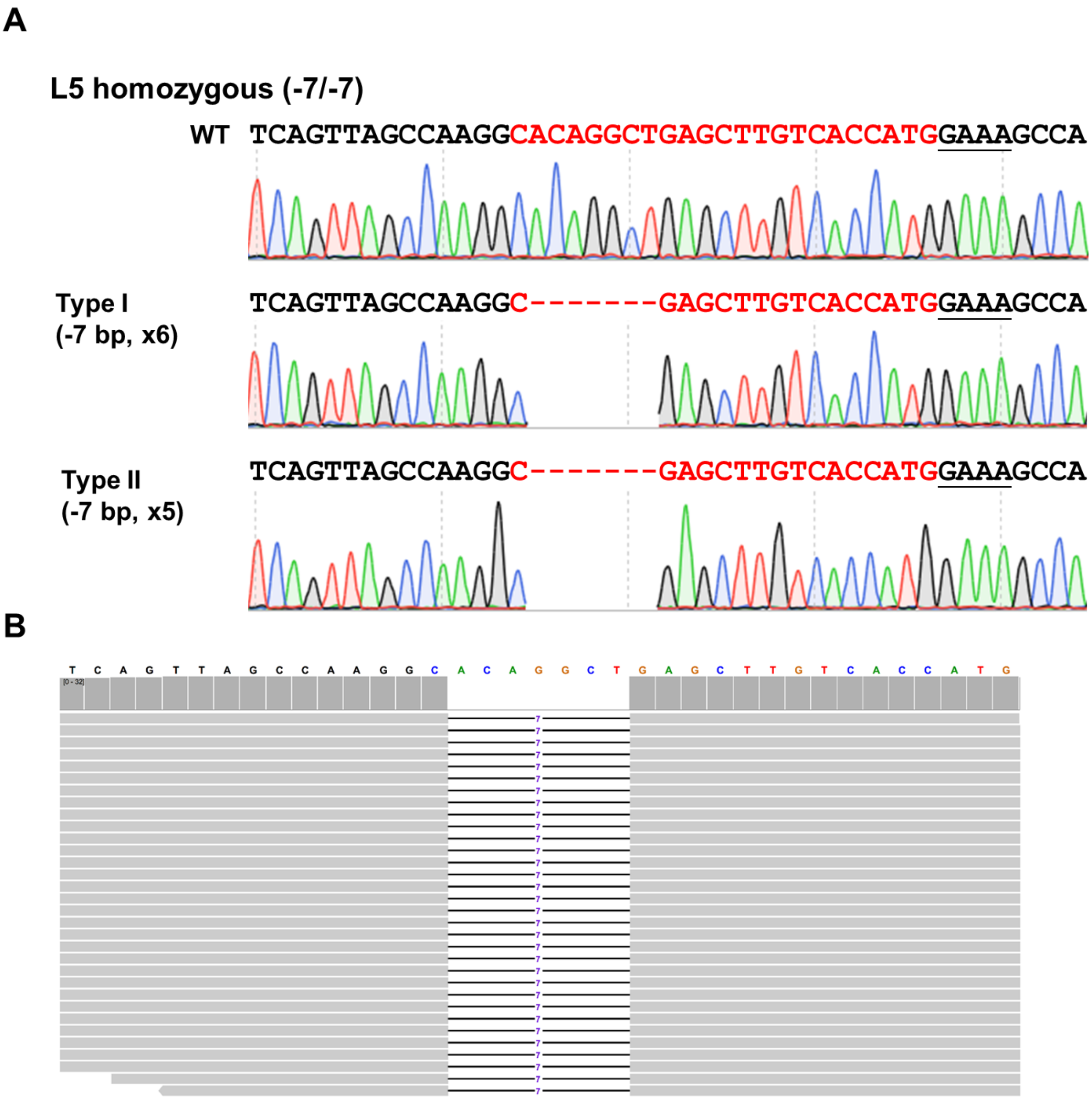

Supplementary Figure 6. Sequencing confirmation of the transgene-free *CsLOB1*-edited *C. sinensis* cv. Hamlin line L5 generated by LbCas12aU/crRNA RNP transformation of embryogenic citrus protoplasts. A. Sequencing confirmation of *CsLOB1* based on PCR amplification and cloning. The representative chromatograms of *CsLOB1* edited *C. sinensis* cv. Hamlin lines. The mutations of both alleles of *CsLOB1* were shown for each line. x indicates number of colonies sequenced. Nucleotide in red indicates crRNA. The underlined GAAA indicates protospacer-adjacent motif (PAM). -: deletion. +: insertion. B. Whole genome sequencing of the edited lines using next generation sequencing. The bases of target site were highlighted by colors other than black. There was only one type of deletion of target site, 7 bp deletion, which was shown by the horizontal bar chart. The vertical bar chart showed the sequence depth for each nucleotide.

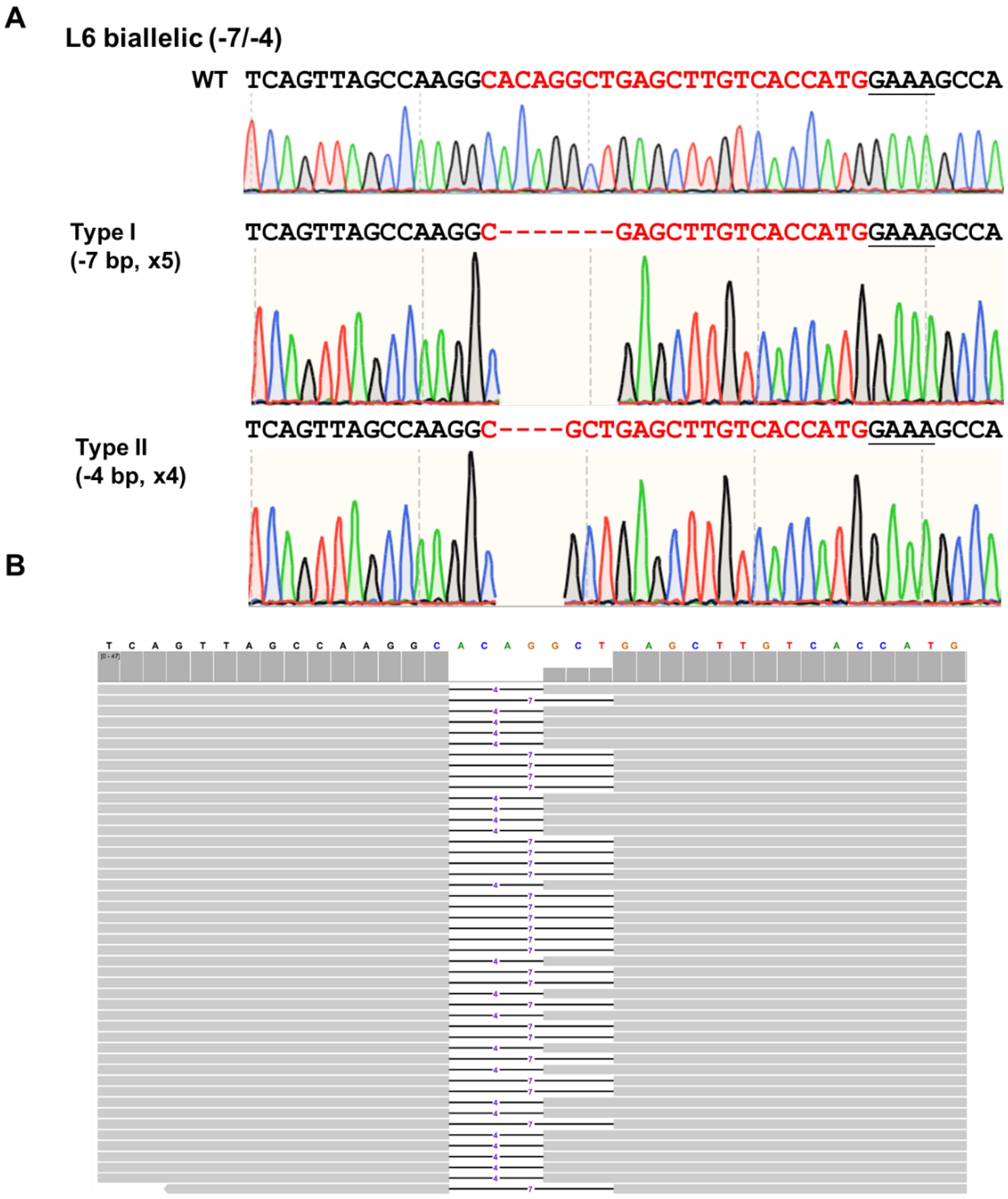

Supplementary Figure 7. Sequencing confirmation of the transgene-free *CsLOB1*-edited *C. sinensis* cv. Hamlin line L6 generated by LbCas12aU/crRNA RNP transformation of embryogenic citrus protoplasts. A. Sequencing confirmation of *CsLOB1* based on PCR amplification and cloning. The representative chromatograms of *CsLOB1* edited *C. sinensis* cv. Hamlin lines. The mutations of both alleles of *CsLOB1* were shown for each line. x indicates number of colonies sequenced. Nucleotide in red indicates crRNA. The underlined GAAA indicates protospacer-adjacent motif (PAM). -: deletion. +: insertion. B. Whole genome sequencing of the edited lines using next generation sequencing. The bases of target site were highlighted by colors other than black. There were two types of deletions of target site, including type I (-7 bp deletion) and Type II (-4 bp deletion), which were shown by the horizontal bar chart. The vertical bar chart showed the sequence depth for each nucleotide.

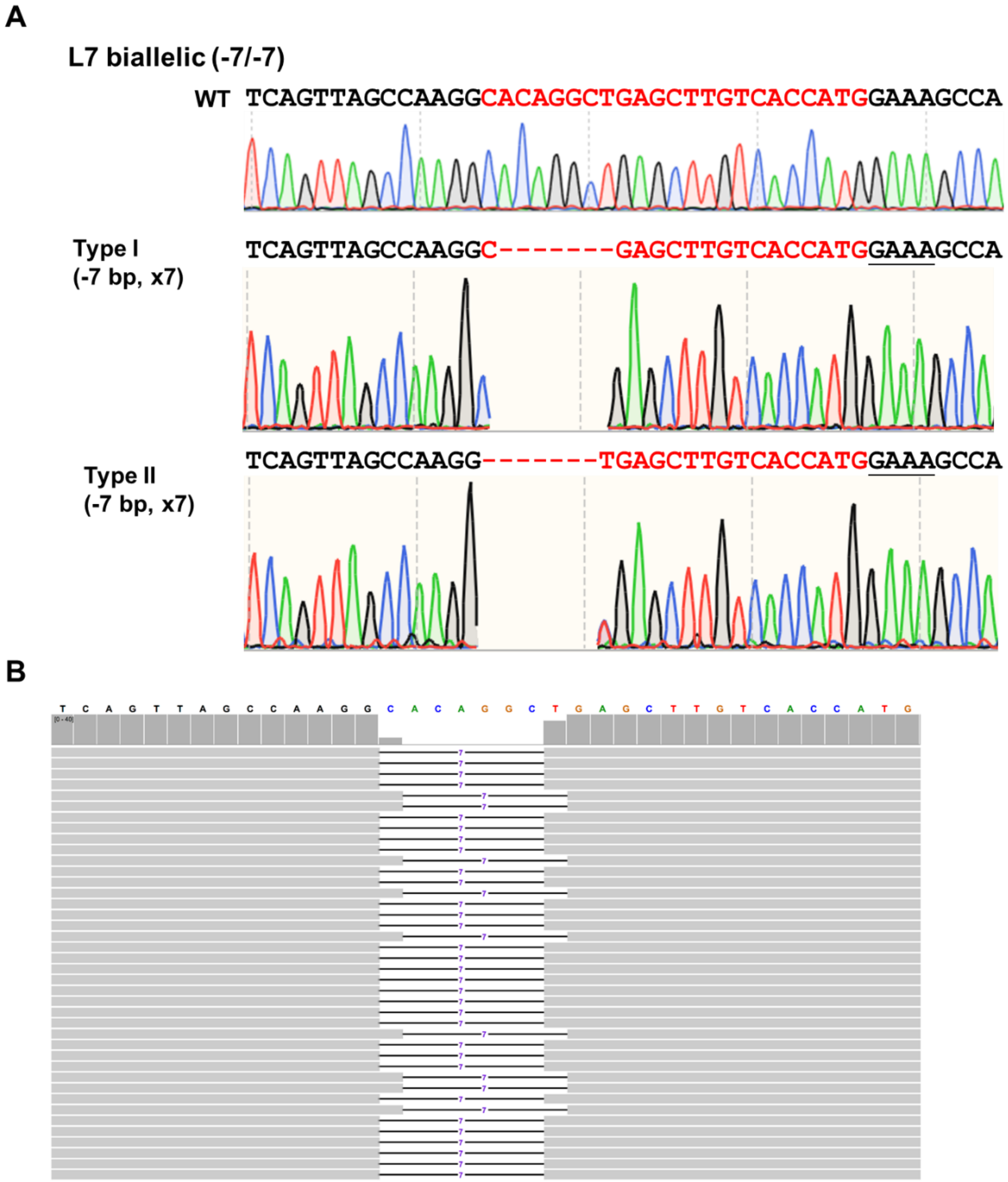

Supplementary Figure 8. **Sequencing confirmation of the transgene-free *CsLOB1*-edited *C. sinensis* cv. Hamlin line L7 generated by LbCas12aU/crRNA RNP transformation of embryogenic citrus protoplasts.** A. Sequencing confirmation of *CsLOB1* based on PCR amplification and cloning. The representative chromatograms of *CsLOB1* edited *C. sinensis* cv. Hamlin lines. The mutations of both alleles of *CsLOB1* were shown for each line. x indicates number of colonies sequenced. Nucleotide in red indicates crRNA. The underlined GAAA indicates protospacer-adjacent motif (PAM). -: deletion. +: insertion. B. Whole genome sequencing of the edited lines using next generation sequencing. The bases of target site were highlighted by colors other than black. There were two types of 7 bp deletions of target site which were shown by the horizontal bar chart. The vertical bar chart showed the sequence depth for each nucleotide.

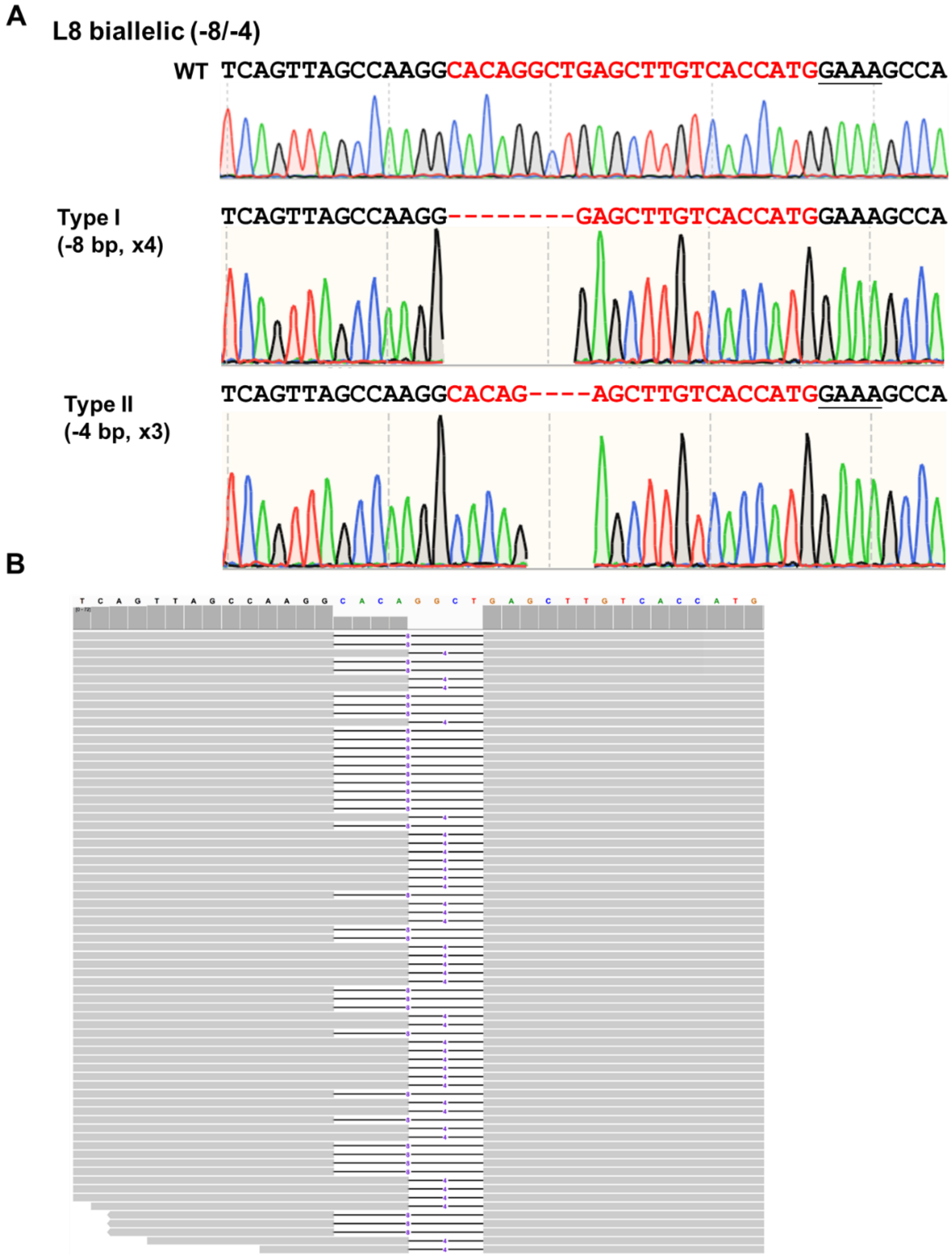

Supplementary Figure 9. Sequencing confirmation of the transgene-free *CsLOB1*-edited *C. sinensis* cv. Hamlin line L8 generated by LbCas12aU/crRNA RNP transformation of embryogenic citrus protoplasts. A. Sequencing confirmation of *CsLOB1* based on PCR amplification and cloning. The representative chromatograms of *CsLOB1* edited *C. sinensis* cv. Hamlin lines. The mutations of both alleles of *CsLOB1* were shown for each line. x indicates number of colonies sequenced. Nucleotide in red indicates crRNA. The underlined GAAA indicates protospacer-adjacent motif (PAM). -: deletion. +: insertion. B. Whole genome sequencing of the edited lines using next generation sequencing. The bases of target site were highlighted by colors other than black. There were two types of deletions of target site, including type I (-8 bp deletion) and Type II (-4 bp deletion), which were shown by the horizontal bar chart. The vertical bar chart showed the sequence depth for each nucleotide.

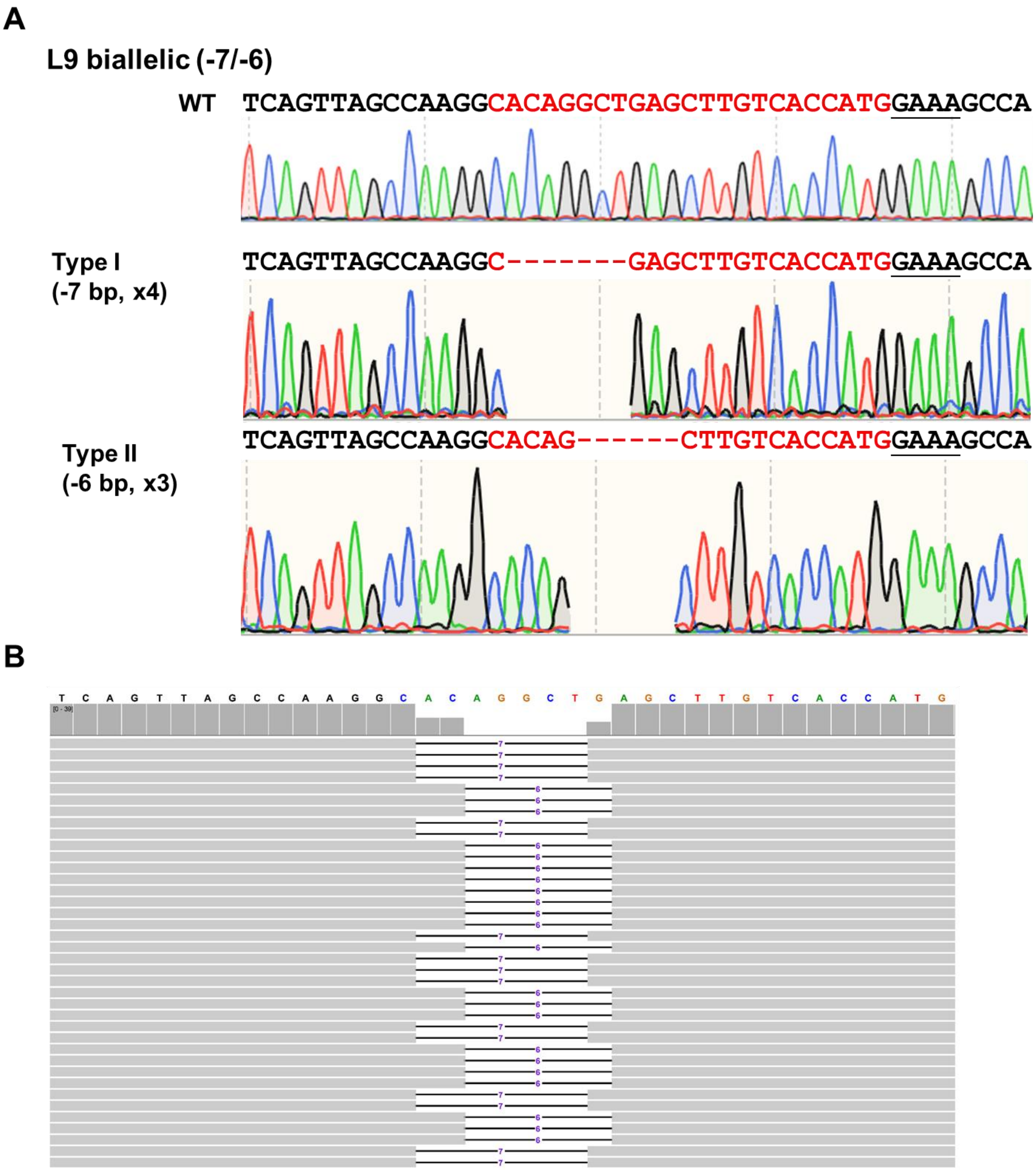

Supplementary Figure 10. Sequencing confirmation of the transgene-free *CsLOB1*-edited *C. sinensis* cv. Hamlin line L9 generated by LbCas12aU/crRNA RNP transformation of embryogenic citrus protoplasts. A. Sequencing confirmation of *CsLOB1* based on PCR amplification and cloning. The representative chromatograms of *CsLOB1* edited *C. sinensis* cv. Hamlin lines. The mutations of both alleles of *CsLOB1* were shown for each line. x indicates number of colonies sequenced. Nucleotide in red indicates crRNA. The underlined GAAA indicates protospacer-adjacent motif (PAM). -: deletion. +: insertion. B. Whole genome sequencing of the edited lines using next generation sequencing. The bases of target site were highlighted by colors other than black. There were two types of deletions of target site, including type I (-7 bp deletion) and Type II (-6 bp deletion), which were shown by the horizontal bar chart. The vertical bar chart showed the sequence depth for each nucleotide.

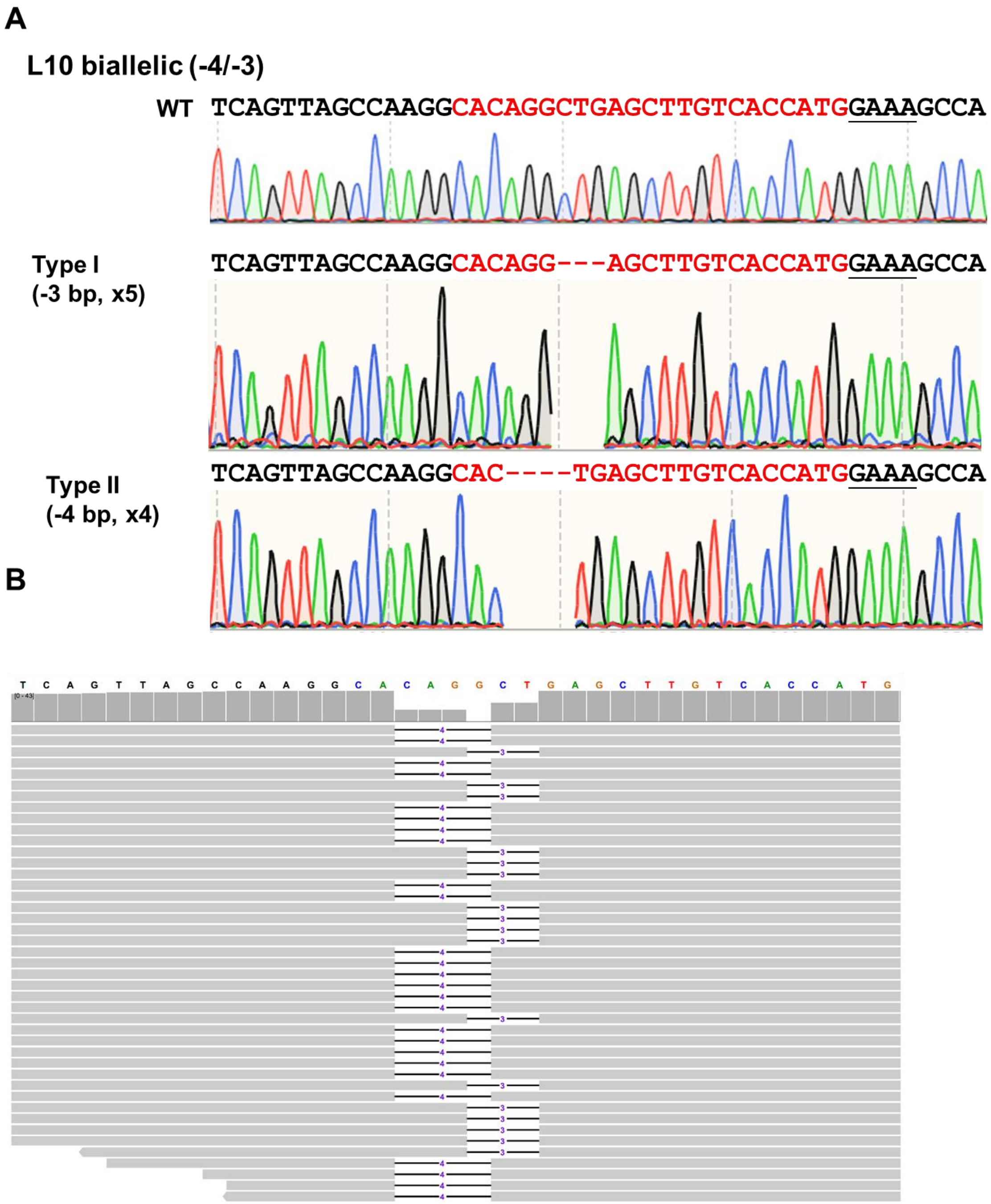

Supplementary Figure 11. Sequencing confirmation of the transgene-free *CsLOB1*-edited *C. sinensis* cv. Hamlin line L10 generated by LbCas12aU/crRNA RNP transformation of embryogenic citrus protoplasts. A. Sequencing confirmation of *CsLOB1* based on PCR amplification and cloning. The representative chromatograms of *CsLOB1* edited *C. sinensis* cv. Hamlin lines. The mutations of both alleles of *CsLOB1* were shown for each line. x indicates number of colonies sequenced. Nucleotide in red indicates crRNA. The underlined GAAA indicates protospacer-adjacent motif (PAM). -: deletion. +: insertion. B. Whole genome sequencing of the edited lines using next generation sequencing. The bases of target site were highlighted by colors other than black. There were two types of deletions of target site, including type I (-3 bp deletion) and Type II (-4 bp deletion), which were shown by the horizontal bar chart. The vertical bar chart showed the sequence depth for each nucleotide.

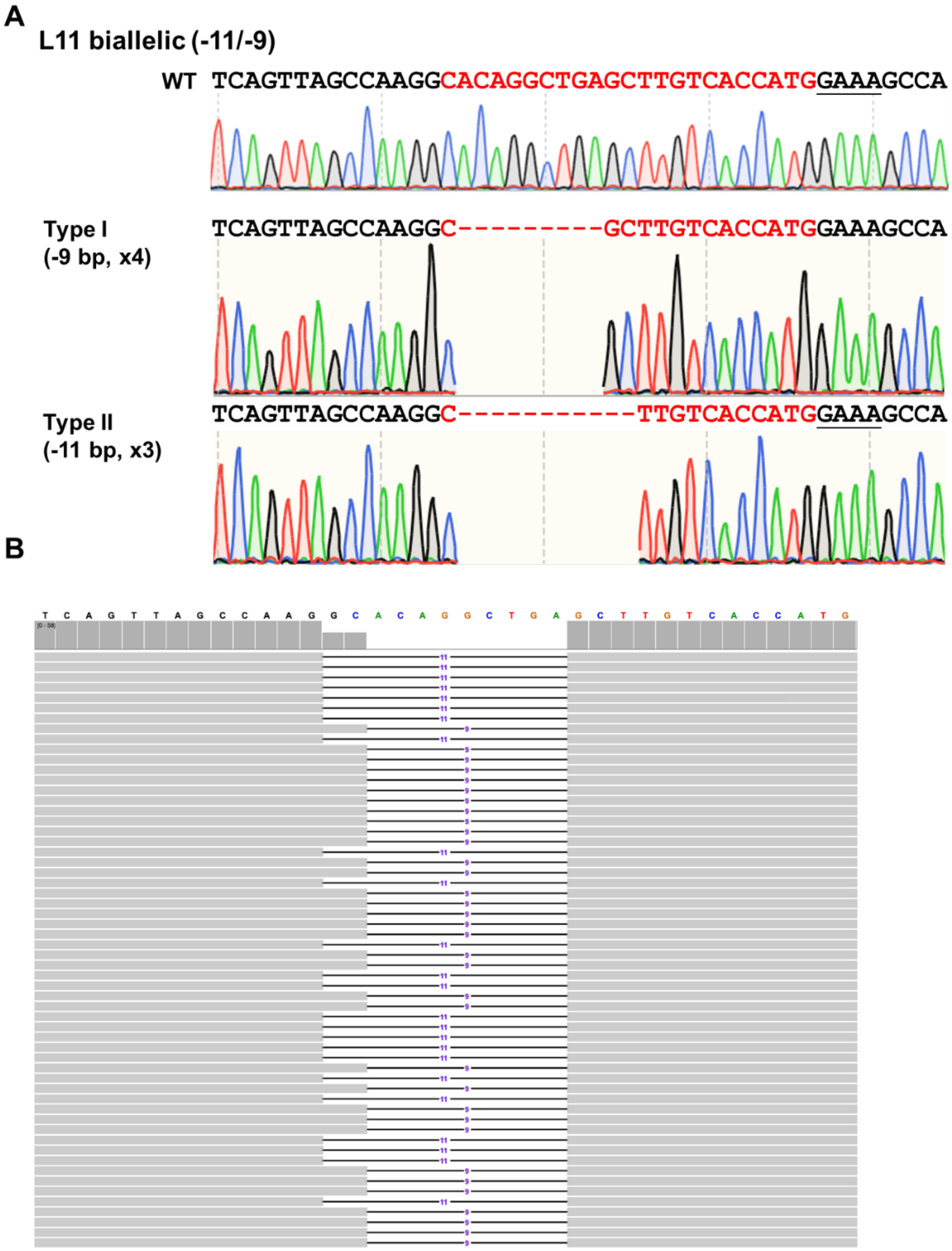

Supplementary Figure 12. Sequencing confirmation of the transgene-free *CsLOB1*-edited *C. sinensis* cv. Hamlin line L11 generated by LbCas12aU/crRNA RNP transformation of embryogenic citrus protoplasts. A. Sequencing confirmation of *CsLOB1* based on PCR amplification and cloning. The representative chromatograms of *CsLOB1* edited *C. sinensis* cv. Hamlin lines. The mutations of both alleles of *CsLOB1* were shown for each line. x indicates number of colonies sequenced. Nucleotide in red indicates crRNA. The underlined GAAA indicates protospacer-adjacent motif (PAM). -: deletion. +: insertion. B. Whole genome sequencing of the edited lines using next generation sequencing. The bases of target site were highlighted by colors other than black. There were two types of deletions of target site, including type I (-9 bp deletion) and Type II (-11 bp deletion), which were shown by the horizontal bar chart. The vertical bar chart showed the sequence depth for each nucleotide.

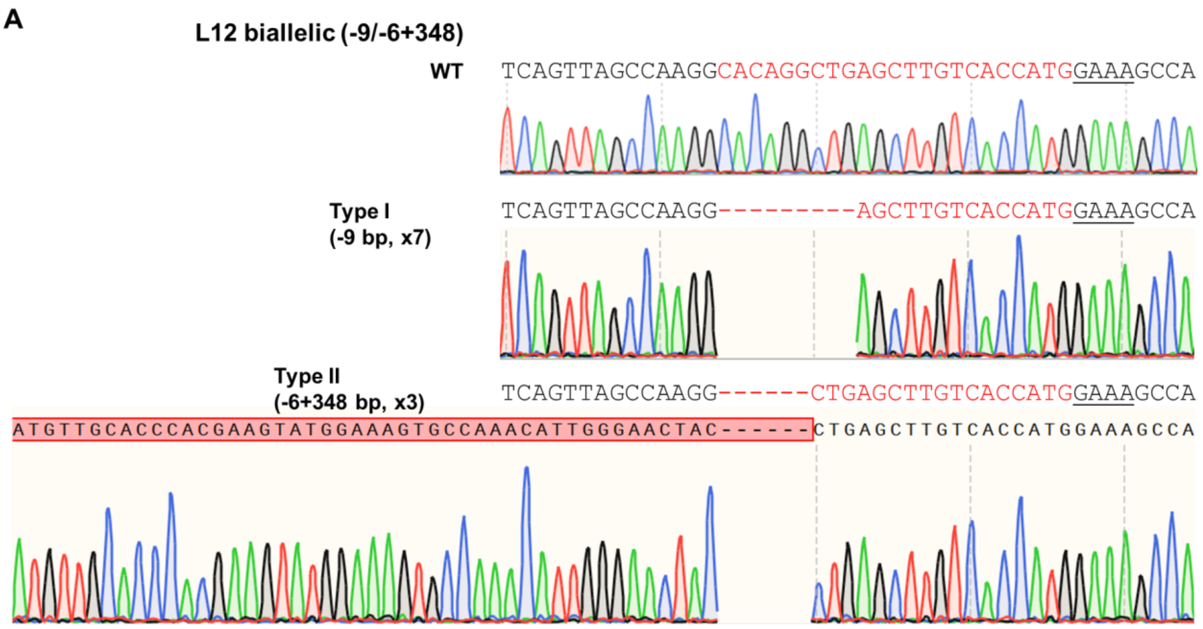

B

```
CCTTGAAAAATTCATATTAACGTTATCAATGATTTTTTTTTAATAGTTTACCACTTATTTTTTTATAACACCTTGGTAATTTTGACATTAGGTAGCAATATAATACGA
TAAAATTCACCTCCATGTAATTTGAAGTTCTTTTCAATAATTTTTTTGACAAATTTTATAGAAGAATTTAACCTTTTTTTTTTTGGTTCAAACGAAGAAATGTTTCCGT
CATTCAATTAAAATTAATGACATCATCTAGTGGCTCGGTGACATACGCTTTAGATACAATTGTCATTCTTGCCTTTTCCTTTCTCTATATAAACCCCTTTTGCCTTG
AACTTTGTTTCAACTAAAGCAGCTCCTCCTCATCCCTTACTGTCTTTGCTTTCTCACTAACTACTACAACCCAACAGTTTTCTTCTCTCAAAAATGGAATGCAAACA
CAAAATTAATGTAGCAATCCCAATCACTAATATGAAGAACACTCAATTCTCATCTCCATCTACTTTCTCTACTTCTCCTCCTTCTCAATCTTCTCCACGCTTCCCTT
CTCCTAATCATCAACAATTGTCTTCTCCAGAATCTTCTCCAAGCTTTAAAGCTTCTCCTTCACAATCCTCTCCAAATCTTGCAGCTCCCCTCTCTCCGCCGCCTAT
AGTTCTTAGCCCTTGTGCTGCTTGCAAAATCCTCCGCCGCAGATGCGTCGAGAAATGTGTTTTAGCTCCATATTTTCCACCAACCGAACCATACAAGTTCACCAT
TGCTCATAGAGTCTTCGGTGCTAGCAATATCATCAAGTTCTTGCAGGTATGCACTTCTTTTGTATGTGATAAATTCAAACTAATTAAATGTCCAACCATTTTTTTTC
TAATTGGGAGAAAAAAAAAACTTGTTAATTGTTTTATTTTCATCAATTAGTTGTGTGATTAGACTTTGGAGTGGTTGATTGTTCCACTCTTTTTTGGAAACTTACGG
ACTTCTCTAATCAAAAGAAAAGAGAGTGTGACATTTCAACTGATTTCATGCAACTTTAATGTTTGTTTTCAATTCACTTTCTTTAATTTAAATAGAGTAGATTATTCA
AATGGATCCTTTTTATTTCTCCTTATAAAGGAATCATCATCACTCTTCCTTGACGGGGCACTGGCGCAGGGTCAATCTGCCTCGTATACAAGTTTTACAAAGCTT
AAATTTTGCCCCTTTCTAGAAAATATTGTGAATTTTACAAAGTCAAACAATGAGTATGAACTTAGCACATGGGTCCACATTAGAAACCAATTCCATGCCCCAGCAG
CTTAATATCATAAAGTTAGCACATGGCCCGTTAAGACTTAAGCTTTGACAGAATCCAGCGCTCAGAGAACGGTTTAAAATGTTTCTCATTTAGACTAGACTTGCA
AATTAAAATTAAAATATTTCAGTCATTTTATTTTATTAAAATGTAAGGGAGGGTAAATCCCAAATTAATTTTTCGCCTCCCATCTTCTCAAGATTCAAATTCTGGTG
GTCAGACAATAAAATTGTTTGAAAACGATTTGAAGCAGCCTTGGGGCTGAAAATTTCAGTCATCCATATTTGCACACAAACAATGTCTCATGCCATTAAAAATTTT
CCAGGAACTGCCAGAATCTCAACGAGCAGATGCAGTGAGCAGCATGGTCTATGAAGCAAGTGCCAGAATCCGGGATCCTGTTTACGGCTGCGCCGGGGCTAT
TTGCCATCTCCAGAAACAAGTCAGTGAGCTTCAGGCTCAGTTAGCCAAGGAAATTCTGGGGTGGTCATTATAGCGGTTCCTTCTGATCCTAGAAAACGTCCGAA
AAAACCTGCTACGGAACTACCTAGCAGGGGCAAAAATACGATAAGTAGATACATAATTTCGAGTGTGATCAGACAACCAAAAATCAGACAATGAGAGAGCGG
CTGAGTGATAGATTGATCGACGTTCGGAGAACGCTCGACCGAAGGAATTTTGCACAAGGTGAATTGTAAGCCCCAACGACTTCGGAACGAGCATTTTCGGGG
ACTAGCCCGCTTAGGTTGTAACTTGATCATGAGCGAATTTGTTGATGTTGCACCCACGAAGTATGGAAAGTGCCAAACATTGGGAACTACCTGAGCTTGTCAC
CATGGAAAGCCAGCAACGCAATTTAATAACTCTAATTTGCATGGAAATGGCACAATCTCAAGAACAAGTCTTGCAGCAGCAGCAGCAGCAGCAGCAACAGTTCA
TGGATACTAGCTGTTTTTTGGATGACAATGGTATTGGATCAGCTTGGGAGCCTCTGTGGACATGATCAAGAGAAATTAAAGCAAGATTGTTGAAATTTTAACCTT
TTAAGAGATTATTTACATAAAGCTAAACATACTTAATTATAAAAGTTTCTGATCAATAATTAAAGTTATTTGCTGCGCGGTAGATGGGAGTGATTATTATGTGCTTT
AATTTTCATTAGTCTTGTTGACAAAAAGGAATCTTTGAACCATCTGGAGAAGTCCTTTGTTAACGGTTCGAGATTAATTATTAGTTTATCTTTATTTACATTTAGTG
AAATTTTGTTTTTAACTAATTTTATAGACATAAATAACCCAACCAAGATGGGAATTCAGTGC
```

Legend: Exon crRNA PAM site insertion sequence

Supplementary Figure 13. Sequencing confirmation of the transgene-free *CsLOB1*-edited *C. sinensis* cv. Hamlin line L12 generated by LbCas12aU/crRNA RNP transformation of embryogenic citrus protoplasts. A. Sequencing confirmation of *CsLOB1* based on PCR amplification and cloning. The representative chromatograms of *CsLOB1* edited *C. sinensis* cv. Hamlin lines. The mutations of both alleles of *CsLOB1* were shown for each line. x indicates number of colonies sequenced. Nucleotide in red indicates crRNA. The underlined GAAA indicates protospacer-adjacent motif (PAM). -: deletion. +: insertion. For the L12 genotype, one allele contained both 6 bp deletion and 348 bp insertion of *C. sinensis* mitochondrial sequence. Only part of the insertion was shown. B. One allele of *CsLOB1* sequence in L12 showing insertion sequence and 6 bp deletion (CACAGG).

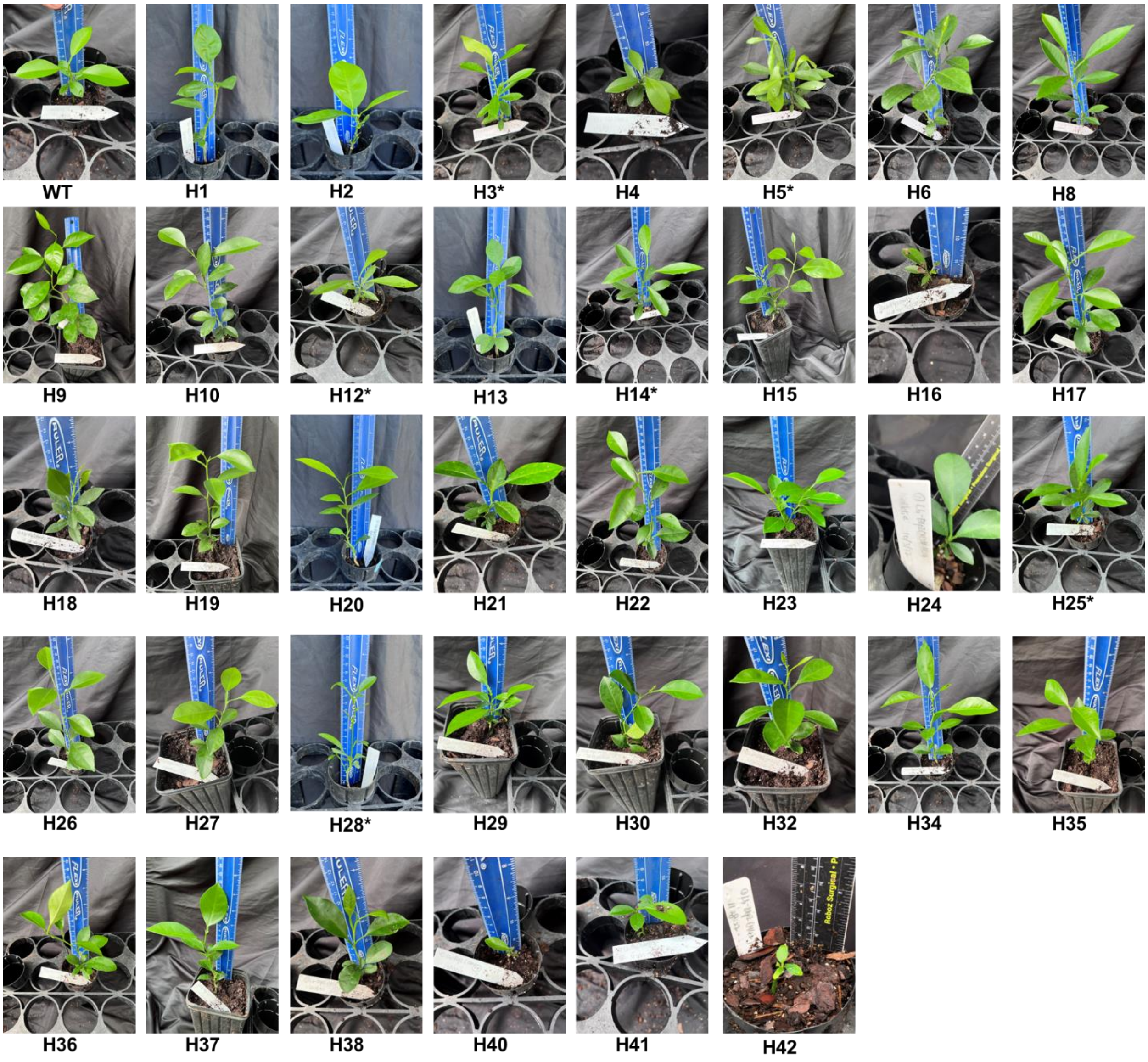

Supplementary Figure 14. Pictures of transgene-free *LOB1* edited *C. sinensis* cv. Hamlin. WT: wild type. Wild type *C. sinensis* cv. Hamlin was generated from seeds and grafted on Carrizo citrange. The edited lines were also grafted on Carrizo citrange. * indicates edited lines showing narrow leaves.

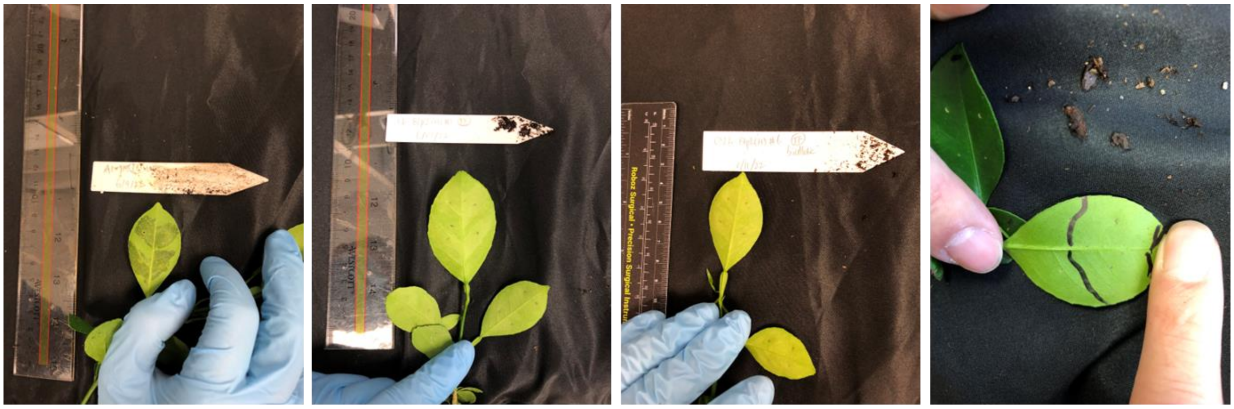

WT + Xcc | ***cslob1* + *Xcc* L1, -7/-11** | ***cslob1* + *Xcc* L3, -7/-14** | ***cslob1* + *Xcc* L5, -7/-7**

Supplementary Figure 15. Canker symptoms on wild type *C. sinensis* cv. Hamlin and *cslob1* mutants. Fully expanded citrus leaves were inoculated with *Xcc* at 10^7 CFU/mL using needleless syringes. The picture was taken at 9 days after inoculation.
